# Commentary: Down with Retirement: Implications of Embodied Cognition for Healthy Aging

**DOI:** 10.3389/fpsyg.2017.00599

**Published:** 2017-04-21

**Authors:** Alex A. Miklashevsky, Martin H. Fischer

**Affiliations:** ^1^Laboratory for Cognitive Studies of Language, International Center for Human Development, Tomsk State UniversityTomsk, Russia; ^2^Division of Cognitive Sciences, Department of Psychology, University of PotsdamPotsdam, Germany

**Keywords:** child development, embodied cognition, lifespan, mobile phone, tablet computers, digital technologies

Hommel and Kibele (2016) proposed a clear model of understanding age-related cognitive effects in elderly people. They claim that, from an embodied cognition perspective “cognitive processes and functions should not just be taken as a given <…>, but as abilities that emerge from active exchange with one's physical and social environment” (p. 2). They model this exchange with the environment as a vicious spiral: elderly people tend to reduce interactions with their environment, which in turn reduces their cognitive abilities further.

This insight seems to be productive not only for discussing cognitive aging but also for any investigations of cognitive development from a lifespan perspective. Particularly, it is interesting for studying dramatically changed childhood development in the light of pervasive digitalization. Recent evidence (reviewed by Kucirkova, 2014; Radesky et al., 2015) indicates considerably increased use of smartphones and tablets by children and teenagers. While adults now acquire the skills of typing, working with touch-screens and communicating in social nets *after* prolonged engagement in and interaction with the physical world, children get engaged *in parallel* with (or even instead of) it. We argue that this time course difference has consequences for spatial, conceptual, numerical and linguistic development. In particular, cognitive asymmetries will emerge because different proportions of analog and digital sensory experiences will shape cognition differently, eventually creating entirely new cognitive mechanisms underlying seemingly similar mental activities in future adults.

Over 90% of American teens use the internet daily, sending and receiving on average 30 text messages a day (Lenhart, 2015). This trend of replacing oral with typed language ties linguistic representations to manual instead of orofacial activity. Learning of letters with vs. without typing them results in stronger vs. weaker brain activation in letter processing respectively (James, 2010). Similarly, Chinese children who type extensively using pinyin (a Romanization system for standard Chinese) have reduced Chinese character reading skills (Tan et al., 2013). Both observations show how embodied experiences transform conceptual representations.

Language itself develops other cognitive functions, such as concept formation (Vygotsky, 1986) and our ability to generate extensive representations during thinking. Smartphones and tablets seriously reduce hand motor activity and haptic exploration when compared to traditional object interactions (see also Spitzer, 2013). Such diminished spatial exploration will lead to shallower understanding of spatial relationships represented in language. As spatial schemas underlie much abstract thoughts (e.g., about time or valence; Lakoff and Johnson, 1980), changes in abstract thought are likely. Spatial relationships are culturally fixed through conceptual metaphors acquired by each generation anew. We predict that “digital children” will replace haptic with visual metaphors—they will no longer “*grasp the idea*” but hopefully still “*see the point*.” More generally, as digitally mediated interactions increase, our motor repertoire tends to shrink (even if we play Kinect™ or search Pokémons™) and we expect to see a drift from motor to visual simulation in language comprehension. For example, when comprehending “he gives you the pizza” we will rely more on a visualization of the implied scene (Stanfield and Zwaan, 2001; for review see Bergen, 2012) rather than engaging our motor system (Glenberg and Kaschak, 2002; for review, see Fischer and Zwaan, 2008).

Finger counting plays an important role in the development of arithmetical skills in children (Domahs et al., 2012). Replacing the sensory and motor activity of natural finger counting with device-supported learning of numbers destroys each number's unique sensory-motor profile, rendering them cognitively less accessible (Sixtus et al., 2017).

There are important differences between real communication and mere observation of communication (e.g., during video watching). Children hardly (verbally) interact with persons on a screen and rarely receive feedback from them. Roseberry et al. (2014) showed that children learn new words from real-life social interaction and communication (also on Skype, i.e., during an interactive process involving perception of the interlocutor) but not from video training alone. Finally, language is a social tool that allows people to achieve goals with the help of others (Borghi and Binkofski, 2014). In virtual scenarios children mostly interact nonverbally. This eliminates the pragmatic value of language, again making it less “woven into action” (Section 7 of Wittgenstein, 1953; as quoted in Pulvermüller, 2012, pp. 425–426).

Digital technologies became part of our everyday life and children interact with them extensively. We have developed the idea of Hommel and Kibele (2016) that one's cognitive abilities are bi-directionally related with active interactions within one's environment. Recent changes in this environment, such as digitalization, predict potential consequences of these changes for human cognition at different stages of ontogenesis. The same high-level cognitive activities can be performed by means of different underlying cognitive mechanisms in adults and children but due to different early sensorimotor and social experiences. Digitalization of early experiences may abolish or transpose cognitive signatures that hinge on analog experiences in early childhood, such as an extensive haptic repertoire and verbal co-operation. Just as older adults, children already avoid real social and physical interactions, thus creating a cognitive asymmetry that may make them spiral away from the world we wish them to own.

## Author contributions

All authors listed, have made substantial, direct and intellectual contribution to the work, and approved it for publication.

### Conflict of interest statement

The authors declare that the research was conducted in the absence of any commercial or financial relationships that could be construed as a potential conflict of interest.
